# Separation of virgin plastic polymers and post-consumer mixed plastic waste by sinking-flotation technique

**DOI:** 10.1007/s11356-021-15611-w

**Published:** 2021-08-05

**Authors:** Washington Orlando Meneses Quelal, Borja Velázquez-Martí, Andrés Ferrer Gisbert

**Affiliations:** Departamento de Ingeniería Rural and Agroalimentaria, Universitat Politècnica de Valencia, 46022 Camino de Vera s/n, Valencia Spain

**Keywords:** Polyolefins, Recycling, Processing, Density, Concentration, Treatment

## Abstract

The main objective of this research is to separate virgin polymers (PA, PC, PP, HDPE; PS, and ABS) and post-consumer plastic waste from municipal solid waste (MSW) using the sinking-flotation technique. Separation was carried out on a pilot scale in an 800-l useful volume container with 160 rpm agitation for one hour. Tap water, ethanol solutions, and sodium chloride at different concentrations were used as densification medium. Virgin polymers were separated into two groups: low-density (HDPE and PP) and high-density polymers groups (PS, ABS, PA, and PC). Polymers whose density was less than that of the medium solution floated to the surface, while those whose density was greater than those of the medium solution sank to the bottom. The experimental results showed that complete separation of HDPE from PP achieved 23% ethanol v/v, whereas high-density polymers separated up to 40% w/v sodium chloride. Polymer recovery ranged from 70 to 99.70%. In post-consumer recycled plastic waste, fractions of 29.6% polyolefins, 37.54% PS, 11% ABS, 8% PA, 12% PC PET, and PVC were obtained. Finally, cast plates were made of the post-consumer waste to properly identify the polymer type present in the separated fractions.

## Introduction

Plastics have become a crucial part of our lifestyles as they are highly functional, hygienic, lightweight, and inexpensive (Pol and Thiyagarajan 2010; Pol and Thiyagarajan 2010). Therefore, its world production has increased exponentially during the last 50 years (Singh et al. 2017; Gu and Ozbakkaloglu 2016; Bucknall 2020). Mumbach et al. (2019) estimated that the world’s cities generated 1.3 billion tons of solid waste in 2012 and forecast that it will increase to 2.2 billion tons by 2025. One of the reasons for the increased consumption of plastics is the various applications they have to replace traditional materials (Burat et al. 2009), especially ceramics and wood (Lackner 2015). According to Mancheno et al. (2016), the highest amount of plastics adding up worldwide is made from polycarbonate (PC), polystyrene (PS), polyvinyl chloride (PVC), polyethylene terephthalate (PET), polymethylmethacrylate (PMMA), and acrylonitrile butadiene styrene (ABS) (Rahimi and García 2017). All this has made the presence of plastics indispensable in the modern lifestyle due to versatility and low production costs (Al-Salem 2019; Geyer et al. 2017).

However, the problem is that all the plastics generated end up as waste causing negative effects on the environment (Huysman et al. 2017). In addition, the management of plastic waste has not been very successful in recent years, which makes it a challenging project (Sharma et al. 2020; Ferronato and Torretta 2019; Gupta et al. 2015; Vitorino de Souza Melaré et al. 2017; Sharma et al. 2020; Chand Malav et al. 2020; Law et al. 2020). Currently about 80% of plastic waste is sent to landfills (Ayeleru et al. 2020). However, most plastics take hundreds of years to disintegrate when they are dumped in a landfill. Thus, the increasing amount of plastic waste is exerting great pressure on the limited space of landfills (Takoungsakdakun and Pongstabodee 2007) causing improper management negatively affecting the environment (Aljerf 2016). Furthermore, landfill leachate penetrates into surface waters posing a serious threat to the health of nearby residents (Du et al. 2020). In correspondence with the continuous growth of post-consumer plastic waste and the inadequate management of these, there is a special interest in continuing to search for efficient, economic, and environmental alternatives to better manage plastic waste (Bing et al. 2016).

Currently there are various technologies to manage plastic waste. Thus, for example, incineration (Achilias et al. 2007; Guney et al. 2013; Huang et al. 2013) is a widely used alternative to eliminate bulky plastic waste; however, this technique generates a large amount of polluting gases such as CO, SO_2_, NO_2_, HCl, and dioxins (Du et al. 2020; Tue et al. 2016). Second, recycling plastic waste is a promising alternative method that generates less pollution and could be very effective (Chen et al. 2011). Recycling involves separating and identifying plastic waste into individual categories for which there are various techniques and methods (Ruj et al. 2015). A very economical separation technology is gravity separation, and it does not generate pollution to the environment. Gravity separation methods include sink-float tanks, jig, shaking table, cylindrical cyclone media separator, and liquid fluidized bed techniques (Pita and Castilho 2017). In this study, for the simplicity of application, the separation by sinking floating is developed; its use consists in varying the density of the aqueous media used in the dense process. Many authors have used this technique to separate polymers, such as polyolefins (HDPE, LDPE, and PP) (Hu et al. 2013). In addition, the sinking-floating separation technique has been and continues to be widely used as a means of separating plastics, especially those that do not have similar density (Dodbiba et al. 2002; Shimoiizaka et al. 1976; Pongstabodee et al. 2008). Some of the means that have been used to recover plastics have been water, solutions saturated with water with sodium chloride, calcium chloride, and ethanol solutions (Fu et al. 2017).

With the aim of improving the procedure and further enriching the literature on the applicability of the technique, the objective of this research is to present as assignment on the separation of different virgin polymers and plastic waste from post-consumer urban solid waste by means of the sinking-floatation method. In addition, for the separation of polymers and plastics, three aqueous separation methods are used: tap water, sodium chloride, and ethanol. Finally, characteristics of separated fractions of post-consumer waste are evaluated by creating melted plates at several melting temperatures.

## Materials and methods

### Materials

#### Virgin plastics

Polymer samples from the Technological Institute of Plastic, AIMPLAS (Valencia-Spain), were used to separate the virgin plastics. Six different types of virgin polymers were used: high density polyethylene (HDPE), polypropylene (PP), acrylonitrile butadiene styrene (ABS), polyamide (PA), polystyrene (PS), and polycarbonate (PC). The virgin PP and HDPE granules had particle sizes between 3.36 and 2 mm in diameter. Although both polyolefins were white, they presented different tonalities in their coloration; thus, PP was much more transparent than HDPE. The shape of ABS polymer was spherical and yellowish. Likewise, PS displayed a spherical shape, while it had a white-transparent hue. Finally, PC featured a white cylindrical shape, while PA granules presented a rectangular shape and white coloration. Table 1 shows characteristics of the virgin polymers used. Recycled polymer plates are shown in Table 2.

**Table 1:** Standard main characteristics of virgin polymers

**Polymer**	**Code**	**Characteristic**	**Standard**	**Units**	**Value**
PP	BJ750	Density	D1505 ATMS	g/cm^3^	0.910
		MFI (190 °C/21.,6 kg)	D1238 ATMS	g/10 min	28
		Flexural modulus	D790 ATMS	Kg/cm^2^	15.500
HDPE	Lotrène Q TR-571	Density	D792 ATMS	g/cm^3^	0.953
		MFI (190 °C/21.6 kg)	D1238 ATMS	g/10 min	0.020
		Flexural modulus	D790 ATMS	MPa	1300
PS	124 N/L	Density	ISO 1183	g/cm^3^	1040
		MFI (190 °C/21.6 kg)	-	g/10 min	-
		Flexural modulus	ISO 178	MPa	3400
ABS	ELIX ULTRA HH 4115 PG	Density	ISO 1183-1	g/cm^3^	1.070
		MFI (230 °C/3.8 kg)	ASTM D1238	g/10 min	3
		Flexural modulus	ASTM D 790	MPa	2000
PA	PA 6 EXTRUDADA	Density	ISO 1183-1	g/cm^3^	1.140
		MFI (190 °C/21.6 kg)	-	g/10 min	-
		Flexural modulus	ISO 178	MPa	2800
PC	TECANAT PC	Density	ASTM 53479	g/cm^3^	1.200
		MFI (190 °C/21.6 kg)	-	g/10 min	-
		Flexural modulus	ASTM 53457	MPa	2200

**Table 2:** Recycled polymer plates

**Separation solution**	**Concentration g/cm**^**3**^	**Fraction used**	**Plate obtained**	**T**_**e**_ **°C**	**Estimated polymer**	**T**_**f**_ **°C**
Ethanol	0,935	Mix 3	1	134–201	PP	168
		Mix 4	2	110–165	HDPE	137
Sodium chloride	1055	Mix 5	3	176–264	PS	220
	1100	Mix 7	4	180–270	ABS	225
	1175	Mix 9	5	200–300	PA	250
		Mix 10	6	140–265	PC, PET, PVC	145–260

*MFI* melt flow index

#### Post-consumer recycled plastics

As post-consumer recycled materials, plastic waste from solid urban waste (MSW) displayed a certain degree of contamination and deterioration due to labels, inks, impurities, and combinations. The waste analyzed consisted of household packaging such as sheets, bottles, cans, jars, margarine containers, yogurt containers, flowerpots, garden tables, and the like. Samples used were mixed residues of various colors with a non-uniform size and shape.

### Experimental procedure

#### Virgin plastic separation

2.0 kg samples of each virgin polymers were thoroughly mixed in a pilot scale batch in a tank, Ecomini ML-100 with a useful volume of 800 l. The waste was mixed with tap water agitating at 160 rpm for 1 h so that the mixtures were completely moistened avoiding air bubble formation. Polymers with a density lower than tap water (PP and HDPE) floated and emerged in the tank while plastic waste with a density higher than tap water (PA, PET, PS and ABS) sank completely. The polymers that sank and floated were centrifuged at 2950 rpm with an MC-250 centrifuge, to remove moisture from the polymers, impurities such as dust and dirt. Then, polymers were weighed to evaluate their recovery as a function of mass balance.

**Fig. 1:**
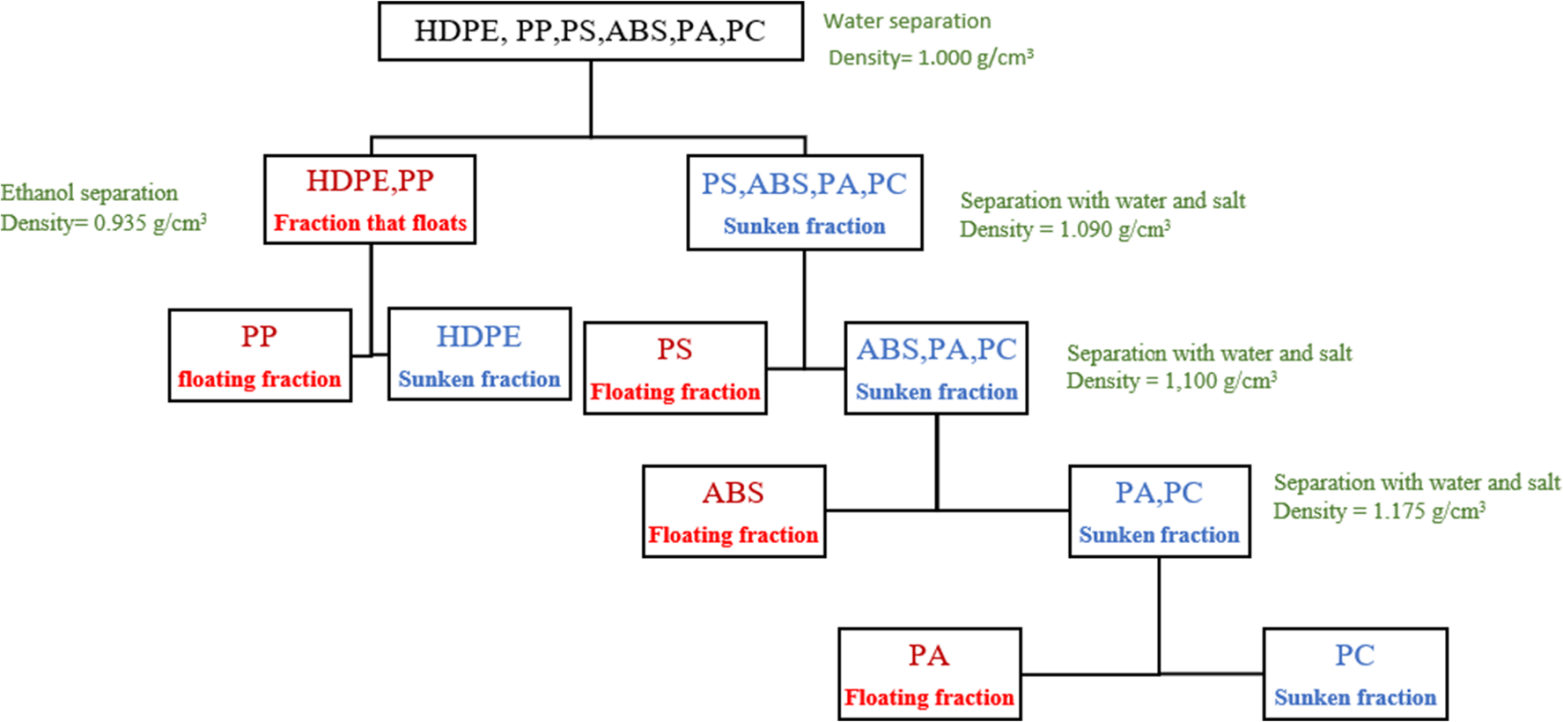
Separation of virgin polymers (HDPE, PP, PS, ABS, PA and PC) in water and NaCl. The polymers written in red are those that float in the solution and the polymers written in blue sink in the solution used

To further separate the polymer mixtures (PP and HDPE) and (PA, PET, PS and ABS) into individual polymers, tap water was replaced by an ethanol and sodium chloride solution. The sodium chloride solution was used with various densities (1.055, 1.100, and 1.175 g/cm^3^), while ethanol was single concentration solution (0.935 g/cm^3^). Figure **1** shows the initial polymer separation process, where a denser polymer than the medium-density sinks to the bottom. Similarly, the polymer with a lower density than the medium-density floats on the surface. Finally, the agitation and centrifugation process in the separation of the polymers with ethanol and sodium chloride were carried out under the same conditions as in the separation with tap water.

#### Separation of post-consumer recyclable plastics

**Fig. 2:**
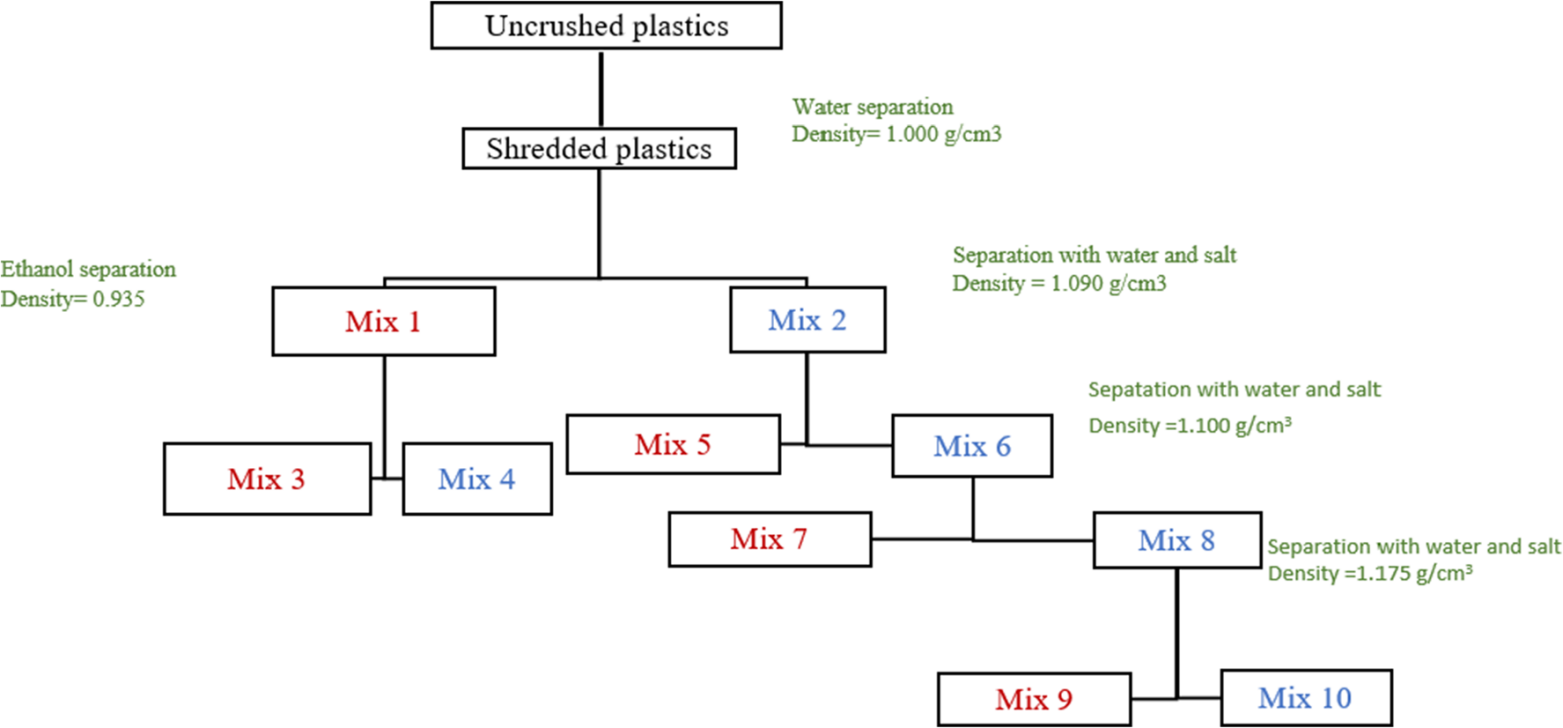
Separation of post-consumer recyclable plastics using tap water, sodium chloride, and ethanol. The mixtures written with red color are those that float in the solution, while the mixtures written with blue color sink in the solution used

**Fig. 3:**
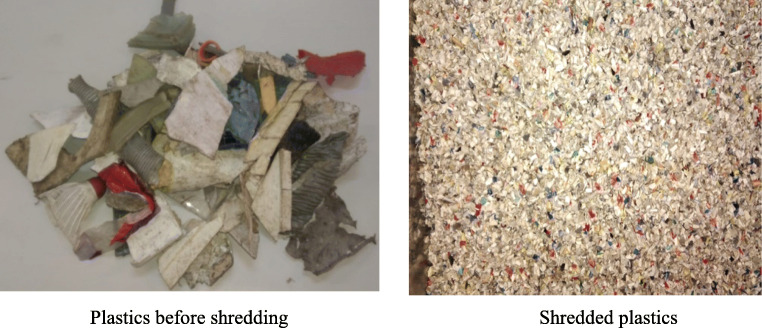
Post-consumer plastics from solid urban waste (MSW) before and after being shredded

Figure 2 shows the process of separating automotive plastic waste with the different solutions (tap water, ethanol, and sodium chloride). Before starting the separation process, plastics were crushed with a RC400 large cutting mill reducing samples size to diameters less than 50 mm. However, to homogenize the samples, size was reduced to 3 mm diameters using a second C17.26 s mill (Fig. 3). In addition, the plastics were of different colors, which facilitated the analysis of their separation. Once all the samples had been crushed (12 kg in total), they were subjected to the separation process with the same conditions (concentration of solutions, tank volume, agitation, and centrifugation) as those used in the separation of virgin polymers. Finally, the compositions and densities of the different mixtures were estimated. The typical densities in the mixtures were determined as an average value of five repetitions.

#### Recycled polymer plates

**Fig. 4:**
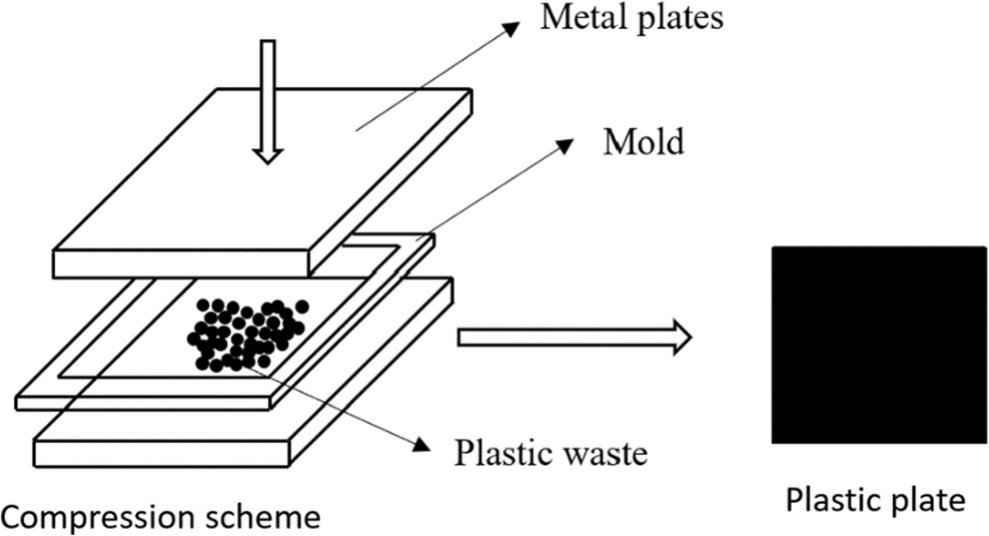
Process of obtaining the plates by means of the compression test

To identify the type of plastic from each separated fraction, plates and sheets were made from each of the fractions. Plates were made by compression using an industrial hydraulic press (FONTUNE PRESES model). The press consisted of two plates, upper and lower, driven by a hydraulic system exerting high pressure. Previously, each fraction particles were distributed in a 20 × 20 cm and 0.2-mm thick mold (Fig. 4). Every plastic fraction, mold, and metal plate was placed in its entirety in the hydraulic press for 10 min at 100 kN pressure with a progressive T_e_, increase in temperature until reaching melting temperature of a fraction. As a reference temperature (T_m_) to melt the separated fractions, the melting temperature of the virgin polymers was used. The temperature used to melt the fractions was between 20 and −20% the melting temperature of the virgin polymer (T_f_), because the fractions were not considered pure polymers since they were mixed with fillers, inorganic additives and mixtures of impurities and irregularities. Subsequently, the temperature was lowered to 60 °C to cool the molten plate. Identifying the type of plastic is contingent on the percentage of ​​the plate’s molten area.

*T*_*f*_ melting temperature of virgin polymers, *T*_*e*_ temperature range of the test

## Results

### Separation of the virgin polymer mixture with NaCl

**Fig. 5:**
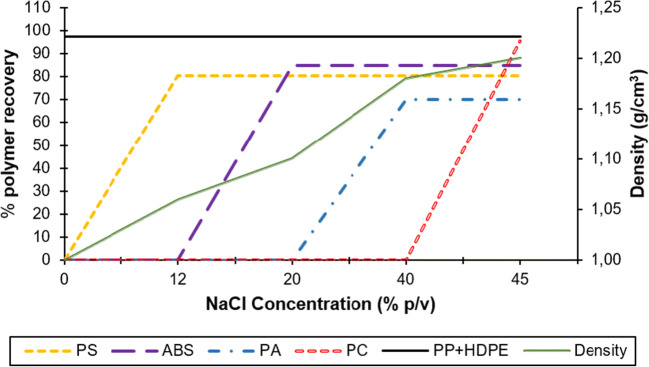
Separation of the PP+HDPE/PS/ABS/PA/PC mixture by the sinking-flotation method with sodium chloride: recovery of the polymers and density of the aqueous sodium chloride solution

In order to separate HDPE, PP, PS, ABS, and PA polymers and PC mixture into individual polymers, an aqueous sodium chloride solution with various concentrations was used (Fig. 5). When only tap water used, 97.5 was recovered % HDPE+PP. However, for an 11–12% w/v NaCl concentration, most PS (80.3%) floated to the surface, while ABS, PA, and PC sank to the bottom. With a further increase in solution concentration (20% w/v), the complete separation of ABS mixture (84.8%) from the PA and PC polymers was achieved. For a 40% NaCl w/v concentration, most of the PA (70%) floated to the surface of the solution, while 95.4% of the PC completely sunk.

### Separation of the virgin polymer mixture with C_2_H_5_OH

**Fig. 6:**
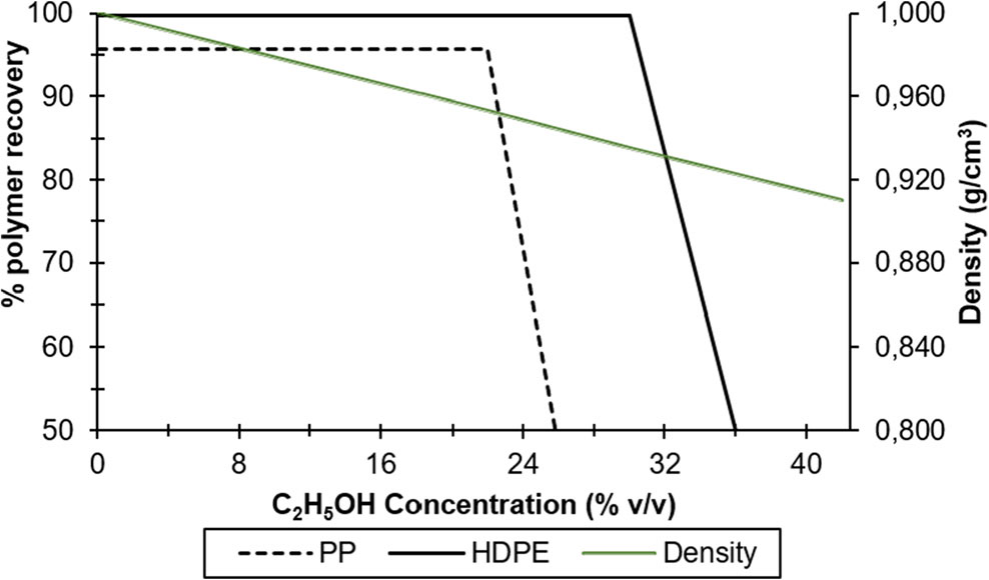
Separation of HDPE from PP by means of a sinking-flotation process with an ethyl alcohol solution: recovery of the polymers and density of the aqueous sodium chloride solution.

HDPE+PP fraction is previously separated with water and ethyl alcohol into individual polymers of PP and HDPE (Fig. 6). The PP, which floated, began to separate from the HDPE when a concentration of 23% v/v of C_2_H_5_OH was used, obtaining a recovery of 95.60%. Complete separation of HDPE from PP was achieved when a concentration of 31% v/v was used. However, the recovery fraction of HDPE was much higher (99.70%). The experimental results demonstrated that the recoveries of HPDE and PP occurred for a density of the aqueous medium of 0.935 to 0.955 g/cm^3^.

### Separation of the post-consumer plastic waste mix with NaCl and C_2_H_5_OH

**Fig. 7:**
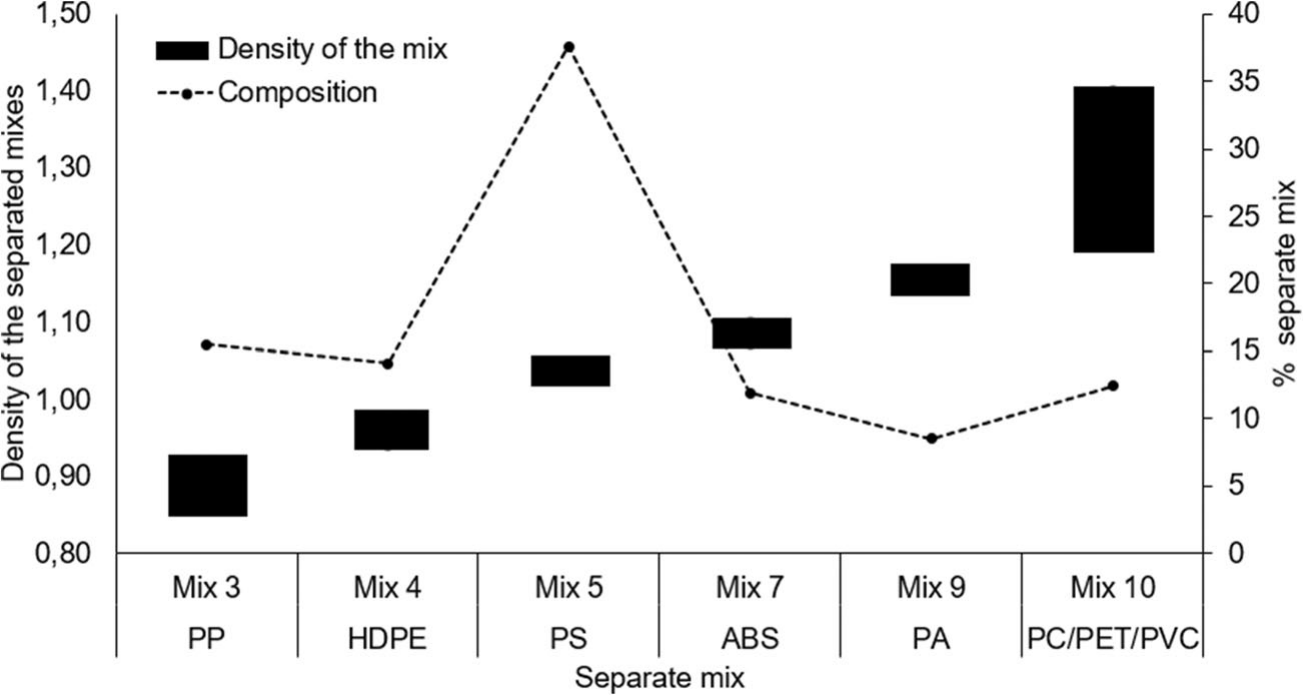
Range of densities and percentage composition of plastic types reported in post-consumer plastic waste from solid urban waste (MSW).

In Fig. 7, six separate MSW plastic fractions are represented. Results showed that plastic waste was mainly composed from mixture 5 (37.5%) and mixtures 3 and 4 with 15.4 and 14.1%, respectively. According to the estimated densities of each separation mixture, the above mixtures could be related to the polymers of PS, PP, and HDPE. To a lesser extent the mixtures 7; 9 and 10 represented 11.9; 8.5 and 12.4%, respectively that could be related to ABS, PA, and PC polymers with some PET and PVC. However, the different fractions are mixed with inorganic fillers and additives, causing properties to vary making the separated samples impure and inconsistent.

### Determining recycled polymers plates

One of the objectives of making the plates is to observe color diversity present in them and determine if the material used melts completely at the polymer melting temperature. If the material melts completely, it means the material has been separated efficiently, that is, the material that makes up this fraction is sufficiently pure without the presence of other polymers, nor impurities and improprieties such as dirt, wood, glass, and metals. On the contrary, if the plate has discontinuous castings or burned areas, it means that the material has not completely melted, causing plate’s heterogeneity due to polymer low purity.

**Fig. 8:**
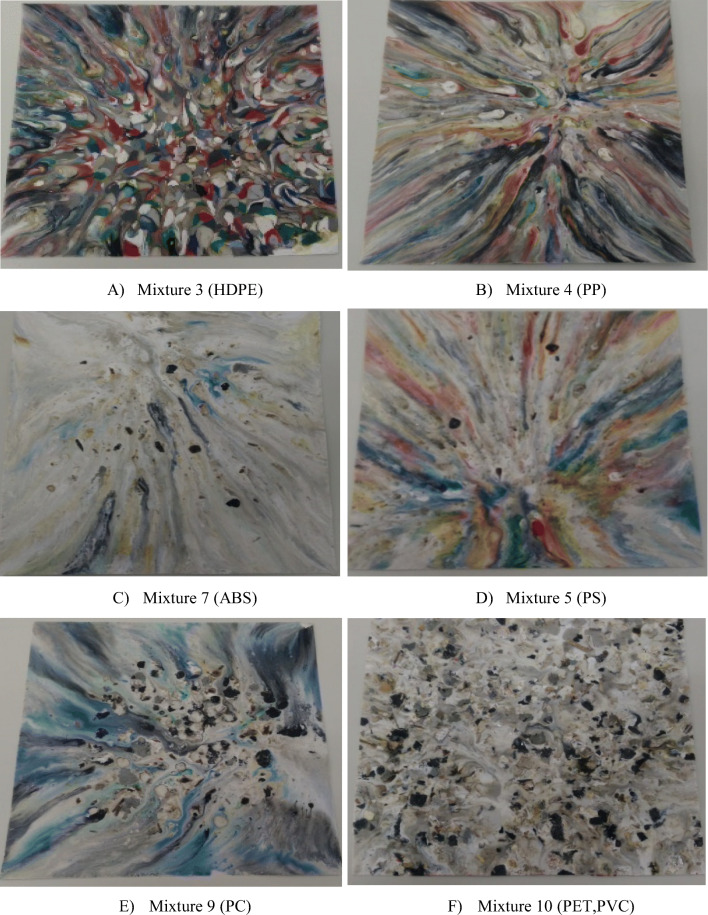
Plates of post-consumer plastic waste melted at different melting temperatures. Mixture 3 was melted at 175 °C, Mixture 4 at 195 °C, Mixture 5 at 220 °C, Mixture 7 at 235 °C, Mixture 9 at 240 °C, and Mixture 10 at 185 °C

Figure 8 shows the plates of post-consumer plastics obtained at different melting temperatures. Thus, for example, mixture 3 was melted at 175 °C, obtaining a high percentage of melting. The fraction did not present cracks and burned areas. However, the separated fraction had brown spots or spots. Stains caused by possible remains of wood and impurities that have prevented its complete melting. Based on the melting temperature and the density of the fraction, this mixture corresponds to HDPE (Fig. 8a). Similarly, mixture 4 melted at 195 °C. The fraction presented few burned areas, denoting a more homogeneous cast, which indicated that according to its melting temperature and density it could be a fraction of PP (Fig. 8b). However, spots of different colors are still noticeable, indicating the presence of other polymers of similar density.

In Fig. 8c, a plate is represented that could mostly contain ABS due to its greater uniformity in its casting; the plate also shows some specific points where it is burned due to the presence of lower density polymers. On the other hand, the plate in Fig. 8d contains less impurity mixture due to the little presence of cracks. However, it has areas that have not completely melted, meaning that there are higher density polymers. It also has some burned parts, indicating the presence of polyolefins and ABS.

Finally, the last two plates of fractions 9 and 10 could correspond to PA, PC, PET, and PVC due to their high densities. The plate in Fig. 8e shows many burned areas and cracks, implying that the purity of the mixture is not optimum because there is a mixture of different polymers. Moreover, the last plate (Fig. 8f) shows brown colors and many black spots because of impurities and improper elements presence (wood, glass soil, remains, and so on.). In addition, this plate has burned areas indicating that it has a lower melting point polymer blend.

## Discussion

### Separation of the virgin polymer mixture with NaCl

Results showed that polyolefins and PC polymers are easier to recover by means of the float-sink technique due to their high recovery percentage. The use of this technique is an interesting alternative to enhance the recycling process. Likewise, ABS and PS groups revealed high recovery percentages, while PA sample separation was not appealing since recovery percentages were quite low. The role played by medium density in the sinking-floating separation technique is a key parameter to separate plastic particles (Fu et al. 2017). For example, PE and PP, as lightweight plastics, can be easily separated by density sorting in the medium of water, while PVC and PET, as heavy plastics, are considered unmanageable plastic blends due to their similar density (Wang et al. 2020). In this study, separation with tap water made the polyolefins separate easily, since these polymers have specific densities lighter than those of water (Ito et al. 2010). The recovery data for polyolefins was higher than 97%, results very similar to other studies in the literature. Thus, Gundupalli et al. (2017) through laser-induced degradation spectroscopy obtained 80–97% polyolefins recovery. Meanwhile, Wang et al. (2015) through foam flotation recovered PE and PP percentages higher than 97%. Similarly, Serranti et al. (2015) through the magnetic density separation technique managed to recover more than 94% of polyolefins. Bonifazi et al. (2013) using hyperspectral imaging technology (HSI) obtained PE and PP recoveries higher than 96%.

Other fractions that had high separation percentages were PS and ABS polymers. Obviously, these polymers possess an inherent buoyancy in higher density aqueous medium, making them manageable for recycling (Wang et al. 2016). Hence, there have been recoveries of over 80%. The high recovery of PS and ABS suggests the presence of an inherent hydrophobic surface of the tested polymers (Du et al. 2020). PA (70%) was the polymer that recovered the least. Their separation could be improved if a selective wetting of polymers was carried out, which allowed a reduction in surface tension (Wang et al. 2015; Alter 2005; Fraunholcz 2004). It should be considered that in this study a natural flotation of the polymers was carried out. One way to increase the recovery of PA is to homogenize the size and shape of the particles. It has been proven in the literature that bar, elongated or irregular particles have greater buoyancy than angular and round particles in the same size range. Furthermore, fine particles float faster than coarse particles (Shen et al. 2001). However, the separation results were very consistent and much better than other previous studies. Qu et al. (2020), for example, through a natural flotation of ABS and PC, they achieved separation percentages of 68.41 and 59.4%, respectively. Similarly, Pita and Castilho (2016) through the gravity jigging method, separated PS from PET and PVC and obtained separation efficiency ranges of 71–85%. For their part, Tsunekawa et al. (2005) through the “Jig” gravity method separated plastics from discarded photocopying machines and managed to recover grades of 99.8% PS, 99.3% ABS, and 98.6% PET.

### Separation of the virgin polymer mixture with C_2_H_5_OH

As it is widely known, both HDPE and PP polymers are naturally hydrophobic. Thus, the set of HDPE+PP samples is easy to separate from the rest of polymers with higher density. However, separating HDPE from PP requires a suitable solution due to their similarity in densities. Therefore, a suitable agent is necessary to achieve an individual selective polymer separation (Kangal and Üçerler 2018). In this study, ethanol as an aqueous solution modifying the separation medium was adequate allowing the two polymers to be separated with a high recovery percentage. However, slight differences in density between HDPE and PP, when separated with ethanol, caused slow sedimentation rates (Ferrara and Meloy 1999).

Additionally, the separation of the mixture between HDPE and PP began with a concentration below 23% v/v of C_2_H_5_OH. Ethanol concentration was much lower than Pongstabodee et al. (2008), who used between 30 and 50% v/v of C_2_H_5_OH to separate HDPE from PP. Nevertheless, in this study, 4.11% additional PP was recovered than HDPE because PP polymer density was much closer than the aqueous medium. Generally, the use of the sink-float separation technique can be very effective in separating polyolefins from each other (Bauer et al. 2018), especially since recoveries close to 100% can be obtained.

### Post-consumer plastic waste separation using NaCl and C_2_H_5_OH mix

In this study plastics were separated into 6 groups based on their estimated density. The obtained fractions demonstrated that the analyzed MSW plastic waste is mainly composed of ABS (mixture 7) and PS (mixture 5). These results are compatible with data from other studies showing that PS and ABS polymers are the most predominant polymers in vehicle waste (Zhang et al. 2020). According to Dahlbo et al. (2018) and Burange et al. (2015), MSW plastic waste is usually composed of PP and HDPE and to a lesser extent by PS (Karmakar 2020). However, variations in the percentage values of MSW plastic waste are usually associated with the consumption habits of the population (Vazquez et al. 2020).

In this research, mixture 10 comprised a 1.20–1.40 g/cm^3^ density implying that the mixture was composed by more than one polymer of the same or similar density. In accordance with estimated density and the percentage of separation obtained, this mixture can be composed by PVC, PET, and some PC remains. In this case, the mixture represented between 8 and 12% of the total composition; values comparable to the estimates of Buekens and Yang (2014), who consider that PET, PVC could represent 10–15% of the waste from a car. On the other hand, PVC and PET plastic waste have similar density levels preventing them from being separated by sinking-floating. PET density changes from 1.33 to 1.37 g/cm^3^ and PVC density is between 1.32 and 1.37 g/cm^3^ (Burat et al. 2009). In other words, the separation of plastics of equal density is not possible by gravity methods (Hopewell et al. 2009). Hence, in mixture 10 a mixture of PET and PVC has been grouped together and they have not been separated into two different fractions. Furthermore, previous studies have found that a strong alkaline solution of NaOH could destroy the hydrophobicity of one of the polymers (Burat et al. 2009; Kangal 2010), preventing the separation by sinking-floating. However, to improve its reproducibility, it is advisable to combine the sink-float technique with other separation techniques to increase the separation efficiency. To optimize the technique, it is advisable to carry it out in stages, that is, to subject the previously separated fractions to a new densification with the same dense medium.

### Determination of plates of recycled polymers

In general, plates contain more than 90% cast, as a result they melted above virgin polymers melting temperature because of the presence of inorganic additives, fillers and possible foaming agents as it was reflected by burned and cracked areas generated during combustion. The fact that the plates have undergone this type of reactions is due to the fact that the foaming material is usually composed by a large number of small foam holes often known as porous polymeric material (Jin et al. 2019). In addition, polymer foam is widely used and plays an important role in the automotive industry (Zhang et al. 2018; Li et al. 2018a, b; Wang et al. 2017; Wang et al. 2018; Li et al. 2018a, b). The results also revealed that the plates presented a variable homogeneity of the polymers, which indicated the presence of more than one polymer in each mixture. This is because the foamers cause the density of the mixture to decrease, causing the plastic material to vary its density.

## Conclusion

This study includes virgin polymers separation by sinking-flotation (HDPE, PP, PS, ABS, PA and PC) and mixed plastic waste from municipal solid waste. The sink-float method with water was highly effective in separating HDPE and PP polymers as up to 97.5% was recovered. Separation of individual HDPE and PP fractions occurred as concentrations of 23% v/v of ethanol were used, obtaining 96% HDPE and 99.7% PP recoveries. Likewise, higher density polymers (PA, PS, ABS, and PC) separation results turned out promising, since recoveries of 70–85% approximately were obtained. Sodium chloride concentration used to separate the polymers was 11–40% w/v. Finally, as per MSW plastics, 29.6% of separate fractions were obtained for polyolefins, 37.54% for PS, 11% for ABS, 8% for PA, and 12% for PC, PET, and PVC.

## Data Availability

The datasets during the current study are available from the corresponding author on reasonable request.
